# Fluorescence-based primary productivity estimates are influenced by non-photochemical quenching dynamics in Arctic phytoplankton

**DOI:** 10.3389/fmicb.2023.1294521

**Published:** 2023-12-07

**Authors:** Yayla Sezginer, Douglas Campbell, Sacchinandan Pillai, Philippe Tortell

**Affiliations:** ^1^Department of Earth Oceans and Atmospheric Sciences, University of British Columbia, Vancouver, BC, Canada; ^2^Department of Biology, Mount Allison University, Sackville, NB, Canada; ^3^Department of Botany, University of British Columbia, Vancouver, BC, Canada

**Keywords:** chlorophyll fluorescence, non-photochemical quenching (NPQ), Arctic phytoplankton, primary productivity, photosynthetic electron transport rates

## Abstract

Chlorophyll fluorescence-based estimates of primary productivity typically include dark or low-light pre-treatments to relax non-photochemical quenching (NPQ), a process that influences the relationship between PSII photochemistry and fluorescence yields. The time-scales of NPQ relaxation vary significantly between phytoplankton taxa and across environmental conditions, creating uncertainty in field-based productivity measurements derived from fluorescence. To address this practical challenge, we used fast repetition rate fluorometry to characterize NPQ relaxation kinetics in Arctic Ocean phytoplankton assemblages across a range of hydrographic regimes. Applying numerical fits to our data, we derived NPQ relaxation life times, and determined the relative contributions of various quenching components to the total NPQ signature across the different assemblages. Relaxation kinetics were best described as a combination of fast-, intermediate- and slow-relaxing processes, operating on time-scales of seconds, minutes, and hours, respectively. Across sampling locations and depths, total fluorescence quenching was dominated by the intermediate quenching component. Our results demonstrated an average NPQ relaxation life time of 20 ± 1.9 min, with faster relaxation among high light acclimated surface samples relative to lowlight acclimated sub-surface samples. We also used our results to examine the influence of NPQ relaxation on estimates of photosynthetic electron transport rates (ETR), testing the commonly held assumption that NPQ exerts proportional effects on light absorption (PSII functional absorption cross section, σ_PSII_) and photochemical quantum efficiency (F_V_/F_M_). This assumption was violated in a number of phytoplankton assemblages that showed a significant decoupling of σ_PSII_ and F_V_/F_M_ during NPQ relaxation, and an associated variability in ETR estimates. Decoupling of σ_PSII_ and F_V_/F_M_ was most prevalent in samples displaying symptoms photoinhibition. Our results provide insights into the mechanisms and kinetics of NPQ in Arctic phytoplankton assemblages, with important implications for the use of FRRF to derive non-invasive, high-resolution estimates of photosynthetic activity in polar marine waters.

## 1 Introduction

For over two decades, single-turnover active Chlorophyll fluorescence (ST-ChlF) techniques have been used for rapid, non-invasive photochemistry measurements, exploiting the inverse relationship between the photochemical and fluorescence yields of Photosystem II (PSII; Kolber et al., 1998). In applying ChlF measurements, it is important to account for competitive heat dissipation processes, known as non-photochemical quenching (NPQ), which decrease ChlF without an associated increase in photochemistry (Müller et al., 2001). In broad terms, NPQ is upregulated under excess excitation conditions when PSII energy absorption outpaces downstream photosynthesis.

Observable NPQ results from several underlying ChlF quenching mechanisms (Triantaphylidès and Havaux, 2009), whose presence and relative amplitudes vary across taxa and environmental conditions. Rapid energy-dependent quenching (qE) is induced by lumen acidification during photosynthetic electron transport, which leads to protonation of PsbS binding sites on light harvesting complexes (LHC; Lavaud, 2007). The resulting LHC conformational changes increase PSII heat losses, thereby competitively downregulating charge separation. Lumen acidification also promotes de-epoxidation of LHC xanthophyll pigments, converting them to fluorescence quenching, anti-oxidant forms (Fernández-Marín et al., 2021). In some phytoplankton groups, not including diatoms, “slow” NPQ (qT) involves migration of mobile LHC units from PSII to PSI during state transitions to balance incoming excitation energy throughout the electron transport chain (Owens, 1986; Chukhutsina et al., 2014). Finally, long-term quenching (qI) results from photo-inactivation or photodamage, which induces PSII core protein synthesis and repair (Campbell and Serôdio, 2020). Collectively, these and other quenching mechanisms act to mitigate PSII over-reduction and associated damage.

Irrespective of the underlying mechanisms, NPQ directly impacts ChlF-based estimates of PSII photosynthetic electron transport rates (ETR), and low light (<10 *µ*mol photons m^−2^ s^−1^) or dark NPQ relaxation periods are commonly applied prior to sample measurement to minimize these effects (Schuback et al., 2021). NPQ relaxation protocols can be optimized in laboratory experiments with individual phytoplankton species, but diverse NPQ responses across taxa (Goss and Lepetit, 2015; Croteau et al., 2021) and variable environmental conditions (Li et al., 2021) make it difficult to identify appropriate NPQ relaxation time-scales for field measurements. Different field studies have thus employed a wide variety of NPQ relaxation protocols, with dark or low light applications ranging from 5 to 60 min (e.g., Alderkamp et al., 2010; Walter et al., 2017; Schallenberg et al., 2020). The use of lengthy NPQ relaxation times is particularly challenging for continuous, underway sampling, potentially leading to photo-physiological shifts from *in-situ* states, and reduced measurement frequency.

Despite the potentially confounding effects of NPQ on ChlF measurements, the NPQ signal itself provides valuable photo-physiological information. Among oceanographers, there is interest in NPQ as a proxy for the electron requirements for carbon fixation (Schuback et al., 2015, 2017; Hughes et al., 2018), and as an indicator of oxidative stress associated with iron limitation (Ryan-keogh and Thomalla, 2020; Schallenberg et al., 2020). Other environmental factors, including high light, low nutrients and low temperature, can also lead to oxidative stress and influence NPQ expression (Lacour et al., 2020). Such conditions exist across much of the Arctic Ocean, where phytoplankton are subject to extreme environmental conditions. In the Arctic summer, high solar radiation and long daylight hours within the stratified and nutrient-poor surface layer contribute to strong NPQ signatures (Lacour et al., 2018; Lewis et al., 2019). During the summer – fall transition, light availability decreases rapidly and wind-mixing resupplies the surface layer with nutrients (Ardyna et al., 2014). Historically, *in situ* studies of Arctic phytoplankton have focused on the mid-summer season (Matrai et al., 2013). However, on-going climate changes are expected to increase the frequency of Arctic fall-blooms associated with longer ice-free seasons (Ardyna et al., 2014; Lewis et al., 2020), motivating further study of phytoplankton photo-physiological properties outside the mid-summer months.

To date, several field studies have demonstrated the effects of environmental variability on NPQ (Suggett et al., 2009; Gorbunov et al., 2011; Alderkamp et al., 2013; Schallenberg et al., 2020; Croteau et al., 2021), but direct studies of NPQ relaxation kinetics have largely been restricted to laboratory cultures of single phytoplankton species (Roháček et al., 2014; Blommaert et al., 2021) or land plants (Long et al., 2022). In this study, we describe NPQ relaxation kinetics in natural Arctic phytoplankton of varying taxonomic composition across a range of environmental conditions. We also present a mathematical framework to justify short NPQ relaxation protocols for ST-ChlF-based ETR estimates, and provide the first *in-situ* analysis of NPQ effects on derived photochemistry estimates. Our results expand current understanding of NPQ relaxation dynamics in natural marine phytoplankton assemblages, and provide new insight into the effects of low-light acclimation protocols on fluorescence-based primary photochemistry estimates.

## 2 Theory: effects of NPQ relaxation on ETR estimates

To derive ETR from ST-ChlF measurements, photosynthetically available radiation (PAR, μmol photons m^−2^ s^−1^) is multiplied by the functional absorption cross section of PSII ( $\sigma^{\prime }$ _PSII,_ m^2^ photon^−1^, or Å^2^ PSII^−1^), and the fraction of PSII reaction centers open for photochemistry (F’_q_/F’_v_, dimensionless).


(1) $$\mathrm{ETR}=PAR*\sigma_{PSII}^{\prime }*F^{\prime }q/F^{\prime }v$$

The prime notation (ʹ) indicates measurements made under background light, which drives both photochemistry and NPQ induction, decreasing ChlF yields. Lower ChlF yields decrease measurement signal to noise ratios, affecting the statistical quality of derived parameters, particularly $\sigma^{\prime }$ _PSII_ (Schuback et al., 2021). For this reason, samples are often exposed to low light prior to ChlF measurements to allow for re-opening of PSII and NPQ relaxation. Light-regulated terms in Eqn. 1 can then be substituted by dark-regulated equivalent terms for higher confidence ETR estimates (Suggett et al., 2010).


(2) ETR=PAR×σPSII∗F′qFM′∗(FVFM)−1

Importantly, this formulation assumes NPQ has an equal effect upon light absorption (σ*_PSII_*) and PSII photochemical efficiency (F_V_/F_M_). This assumption is based on the definition of σ _PSII_ as the product of the PSII optical absorption area ( $a_{PSII}$ ) and F_V_/F_M_ (Kolber et al., 1998; Kromkamp and Forster, 2003), such that:


(3) $$\frac{\sigma \prime_{PSII}}{\sigma_{PSII}}=\frac{\frac{F_{V}^{\prime }}{F_{M}^{\prime }}*a_{PSII}}{\frac{F_{V}}{F_{M}}*a_{PSII}}$$

From the relationship presented in Eq. 3, it follows that robust ETR estimates can be obtained without extended NPQ relaxation periods,


(4) $$\frac{{\sigma^{\prime \prime }}_{PSII}}{\sigma_{PSII}}=\frac{\frac{F_{V}^{\prime \prime }}{F_{M}^{\prime \prime }}*a_{PSII}}{\frac{F_{V}}{F_{M}}*a_{PSII}}$$

Here, ″ notation implies measurements made under low-light or darkness, when NPQ is only partially relaxed. Under these conditions, samples are not fully acclimated to low light, but the PSII pool reopens for photochemistry and rapidly reversible NPQ components are relaxed, improving data signal to noise. Substituting terms derived in Equation 4, Equation 2 can be rewritten as:


(5) ETR=PAR∗σ″PSII∗F′qFM′∗(FV″FM″)−1

This modified approach does not require full NPQ relaxation, thus enabling rapid measurements amenable to high-frequency autonomous data collection. Such high-resolution measurements are necessary to characterize *in-situ* phytoplankton responses to dynamic environments and fine-scale oceanographic variability (e.g., narrow hydrographic fronts or upwelling plumes).

## 3 Materials and methods

### 3.1 Field sampling

**Figure 1:**
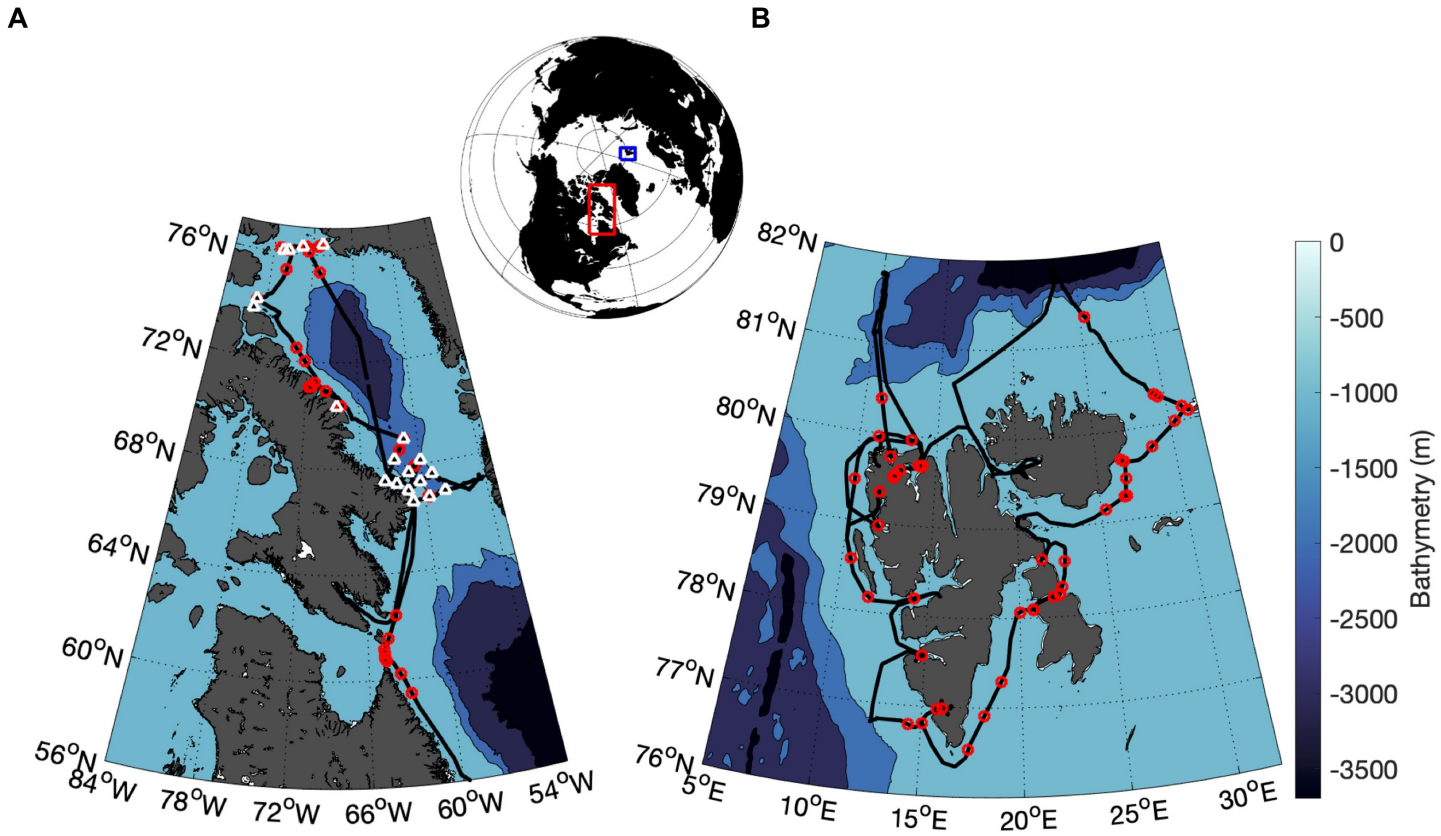
Location of field sampling stations during summer, 2022. Underway NPQ relaxation curves were conducted at locations indicated by red circles along ship survey tracks (black line) in the Canadian Arctic Archipelago, CAA **(A)** and Svalbard Archipelago **(B)**. In the CAA, NPQ relaxation curves were also measured at discrete oceanographic stations indicated by white triangles. Nutrient and pigment data are available for these stations. The CAA and Svalbard sampling regions are shown as red and blue boxes, respectively, on the upper map.

We present results from two ship-based surveys of Arctic phytoplankton assemblages during summer and early fall, 2022. During June 24 – July 7, 2022, surface seawater was collected from an underway supply system aboard the *Le Commandant Charcot* during a circumnavigation of the Svalbard Archipelago (Figure 1). This cruise was operated primarily for tourism, with limited opportunities for discrete scientific sampling, and limited ancillary data. Later in the season, additional discrete and continuous sampling was conducted in the Canadian Arctic (CAA; Figure 1) aboard the *CCGS Amundsen* from September 23 to October 16, 2022, as part of the ArcticNet program. During both cruises, photophysiological properties of phytoplankton assemblages were monitored with a bench-top LIFT Fast Repetition Rate Fluorometer (FRRF; Soliense Inc.) using a single-turnover (ST) flash protocol, as described in Sezginer et al. (2021). Background fluorescence blanks were prepared by gently filtering samples through 0.2 um GF/F filters. Blanks were prepared for each discrete sample, and once daily for continuous underway data, with derived blank values subtracted from all measurements.

Seawater for continuous, underway sampling was obtained from the ship’s surface intake supply, drawn from a nominal depth of ~7 m. A peristaltic pump actuated by Soliense LIFT software was used to draw water into the measurement cuvette in synchronization with autonomous data acquisition routines. The FRRF was programmed to continuously collect 10 successive ST-ChlF transient measurements per sample without background illumination. Between samples, the cuvette was flushed with water from the underway line for 2 min. This underway sampling was interrupted every 4 h by NPQ relaxation experiments (see section 3.2), and by rapid light curves (PvE measurement; data not shown here). In the CAA, additional discrete samples were collected using Niskin bottles from the surface (~ 1 m) and subsurface Chl maxima (10–40 m).

In addition to FRRF measurements, the ship’s continuous seawater line supplied an underway flow-through system for hydrographic measurements on both vessels. The flow-through system on the *CCGS Amundsen* included a Seabird thermosalinograph (SBE 38), and a WETstar fluorometer (WET Labs) for [Chl*a*] estimates. On *Le Commanant Charcot*, temperature and salinity were measured with a SBE 45 Seabird thermosalinograph, but no fluorometer was available.

Underway hydrographic measurements in the CAA were supplemented with on-station CTD casts. Mixed layer depths were calculated with a density-difference criterion of 0.02 kg m^−3^, following Noh and Lee (2008). Niskin bottle sampling was used to calibrate a CTD mounted nitrate sensor (Seabird SUNA) and Chl*a* fluorometer (Seapoint chlorophyll fluorometer). Depth profile data were provided by the Amundsen Science group of Université Laval, and are available from the Polar Data Catalog (10.5884/12713).

On the CAA cruise, we collected samples for photosynthetic pigment analysis, as a source of phytoplankton taxonomic information. For these samples, two 1 L dark Nalgene bottles were filled directly from Niskin bottles from the surface and subsurface chlorophyll maxima depth. Within 1 h of sampling, samples were filtered under low light onto 45 mm GF/F filters (Whatman, 0.7 μ m pore size) and immediately placed in a −80°C freezer. Filters were shipped on dry ice to the Estuarine Ecology Laboratory at the University of South Carolina for high performance liquid chromatography analysis of pigment concentrations. Pigment data were analyzed using CHEMTAX software to identify the relative abundances of phytoplankton groups (Wright and Jeffrey et al., 1997; Higgins et al., 2011). An initial pigment matrix, specific for Arctic phytoplankton was taken from Coupel et al. (2015). To determine photoprotective to photosynthetic ratios (PP:PS) of carotenoid concentrations, the total concentrations of Alloxanthin, Carotene, Diadinoxanthin, Diatoxanthin, Zeaxanthin, and Antheraxanthin were divided by the total concentrations of 19′-butanoyloxyfucoxanthin, fucoxanthin, 19′-hexanoyloxyfucoxanthin, and peridinin.

### 3.2 Photophysiology and NPQ relaxation kinetics

Primary photophysiological parameters (see Supplementary material S1) were derived by fitting the biophysical model of Kolber et al. (1998) to ChlF transients produced by the ST flash protocol. Excitation flashlets consisted of a 25,000 *µ*mol photons m^−2^ s^−1^ light pulses centered around 445 nm with 1 *µ*s duration separated by dark intervals of 2.5 *µ*s. Physiological parameters were monitored during NPQ relaxation curves, which were initiated in freshly collected samples exposed for one-minute to 500 *µ*mol photons m^−2^ s^−1^ irradiance supplied simultaneously by 5 colored lamps (445, 470, 505, 530, and 590 nm), each providing 100 *µ*mol photons m^−2^ s^−1^. Previous work has shown Arctic phytoplankton reach a stable light regulated state within minutes (Sezginer et al., 2021), such that a one-minute high light treatment should be sufficient to produce an NPQ response without compromising sampling frequency. Light saturation values for Arctic phytoplankton range from 50 to 450 (Ko et al., 2020; Sezginer et al., 2021), so we selected a high light treatment of 500 *µ*mol photons m^−2^ s^−1^ to ensure supersaturation. Following high light exposure, a 30 min low intensity, far-red actinic light (5 *µ*mol photons m^−2^ s^−1^, 730 nm) treatment was applied to samples to relax NPQ, following the recommendation of Schuback et al. (2021).

**Figure 2:**
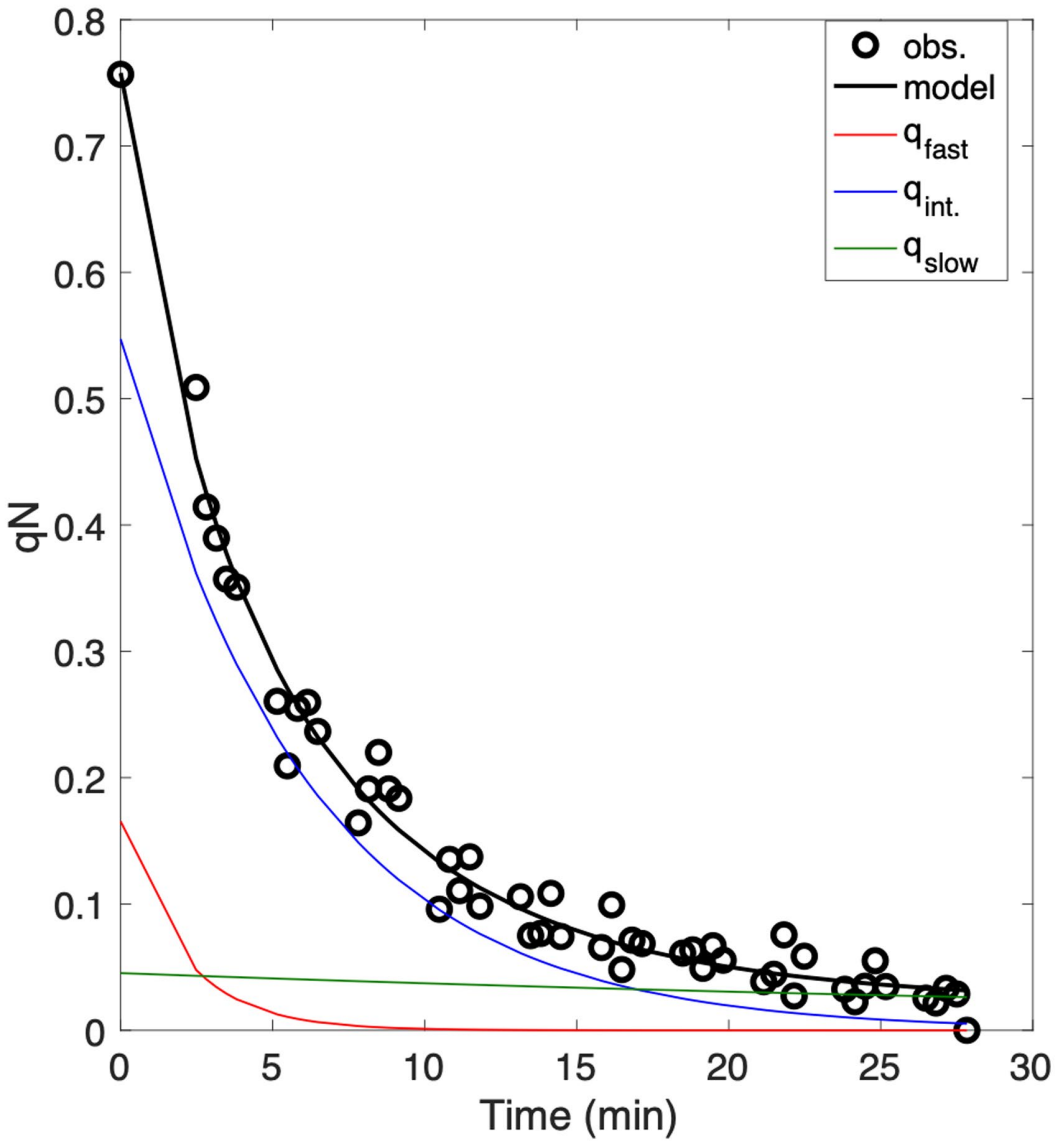
Example of data analysis used to characterize NPQ relaxation time-scales following a one-minute high light exposure. Data points obtained from repeated FRRF analysis are shown as white points, while the black line represents the model fit to the data, combining fast, intermediate and slow components (q_fast_, q_int_, and q_slow_) of NPQ, which are displayed in green, blue, and red, respectively.

During the relaxation period, the coefficient of non-photochemical quenching was calculated as the ratio of quenched to unquenched variable fluorescence, $qN\left(t\right)=1–F_{V}^{\prime \prime }\left(t\right)/F_{V}$ (Roháček, 2010). Total qN is the sum of underlying quenching components. Following the approach of Roháček (2010), we fit a three-component exponential decay model to the observed qN time series to describe NPQ relaxation kinetics and the relative contributions of fast, intermediate and slow quenching (Figure 2):


(6) $$qN(t)=qN_{0}(q_{fast}e^{-\frac{t}{{Τ}_{fast}}}+q_{\mathrm{int}}e^{-\frac{t}{{Τ}_{\mathrm{int}}}}+q_{slow}e^{-\frac{t}{T_{slow}}})$$

The three components of NPQ have relaxation life-times of $\tau_{fast},\tau_{\mathrm{int}},$ and $\tau_{slow}$ . The initial amplitudes of q_fast_, q_int_, and q_slow_ sum to 1, and total quenching immediately following high light exposure equals qN_0_. The total NPQ relaxation life time was calculated as the weighted mean of the individual relaxation life times:


(7) $$\tau_{qN}=q_{fast}*\tau_{fast}+q_{\mathrm{int}}.*\tau_{\mathrm{int}}+q_{slow}*\tau_{slow}$$

For all measurements, curve fits with R^2^ values <0.9 were excluded from further analysis. To avoid overfitting, we applied Akaike’s Information Criterion to compare our three-component model against a two-component model. With few exceptions, the three-component model outperformed the two-component model, justifying the generalized fitting of fast, intermediate and slow relaxation components.

### 3.3 Photosynthetic electron transport rates

Photosynthetic electron transport rates in the presence of 500 *µ*mol photons m^−2^ s^−1^ were calculated according to Eq. 5. The light-regulated photochemical yield of PSII was taken as the average $F_{q}^{\prime }/F_{m}^{\prime }$ measured during the high light exposure period. The PAR term was set to 500 *µ*mol photons m^−2^ s^−1^. Values of $\sigma \prime \prime_{PSII}$ and $F_{V}^{\prime \prime }$ / $F_{M}^{\prime \prime }$ recorded throughout the relaxation period were used to follow any change in ETR as a function of NPQ relaxation.

### 3.4 Statistical analysis

We performed Kruskal-Wallis and multi-comparison tests to compare NPQ relaxation kinetic parameters between CAA surface, subsurface, and Svalbard Archipelago phytoplankton assemblages. Pearson correlation coefficients were used to assess the co-variation in ${\sigma^{\prime \prime }}_{PSII}$ and $F_{V}^{\prime \prime }$ / $F_{M}^{\prime \prime }$ during NPQ relaxation. Spearman rank correlations were used to examine relationships between environmental and photophysiological variables. All curve fitting was performed using least squares methods using Matlab (R2020a).

## 4 Results and discussion

### 4.1 NPQ relaxation kinetics

Across all samples, application of a short, high light treatment (500 μmol photons m^−2^ s^−1^) induced a strong initial NPQ response, with qN_0_ ranging from 0.80 to 1 and showing an average relaxation life-time ( $\tau_{qN}$ ) of 20.0 ± 1.9 min. Significant differences in qN_0_ were detectable between sampling regions. The greatest values of qN_0_ were observed in the subsurface CAA (average = 0.93 ± 0.01), followed by the surface CAA (average = 0.90 ± 0.01), and surface Svalbard (average = 0.88 ± 0.01) samples (Table 1). We observed a positive correlation between the magnitude of qN_0_ and the life time for relaxation (*r* = 0.47, *p* < < 0.01), with the highest $\tau_{qN}$ in the subsurface CAA, followed by the surface CAA, and surface Svalbard samples.

**Table 1:** NPQ relaxation parameters (mean ± standard error) for all study regions.

	CAA subsurface – Sept/Oct (*n* = 19)	CAA surface – Sept/Oct (*n* = 48)	Svalbard surface – June/July (*n* = 58)
qN_0_	0.93 ± 0.01	0.90 ± 0.01	0.88 ± 0.01
$\tau_{qN}$ (minutes)	45.54 ± 4.32	17.76 ± 2.09	13.28 ± 1.42
q_Fast_	0.15 ± 0.01	0.15 ± 0.03	0.11 ± 0.02
$\tau_{fast}$ (minutes)	0.45 ± 0.13	0.59 ± 0.12	0.46 ± 0.07
q_Int._	0.69 ± 0.05	0.81 ± 0.03	0.87 ± 0.02
$\tau_{\mathrm{int}.}$ (minutes)	5.84 ± 2.85	5.83 ± 0.81	5.93 ± 0.50
q_Slow_	0.16 ± 0.02	0.04 ± 0.01	0.02 ± 0.00
$\tau_{slow}$ (minutes)	267 ± 30.6	188± 28.2	217 ± 20
Mean surface PAR (*µ*mol photons m^−2^ s^−1^)	5.0 ± 1.9	76.5 ± 1.3	1,0780 ± 224
PP:PS	0.18 ± 0.01	0.30 ± 0.02 (n = 19)	N/A

The fractional contribution of each quenching component to the total qN_0_ amplitude is denoted as q_Fast_, q_Int._, and q_slow_, while the half-lives of each quenching component are denoted as $\tau_{fast}$ , $\tau_{\mathrm{int}},$ and $\tau_{slow}$ . The ratio of photoprotective to photosynthetic pigments is abbreviated as PP:PS.

Across all sampling regions, the total qN relaxation signal was well described as a combination of three kinetic relaxation components, operating on time-scales of seconds (q_fast_,), minutes (q_int._), and hours (q_slow_.). Variability in $\tau_{qN}$ among samples was driven by differences in the relative amplitudes of the individual quenching components. For example, q_slow_ was minimal in Svalbard samples where qN relaxed fastest. Relative to the Svalbard samples, the contribution of q_slow_ to total qN was two-fold higher in CAA surface samples, and four-fold higher in subsurface CAA samples. The reverse pattern was apparent in q_int_, whose contribution to qN was greatest in Svalbard samples, and lowest in CAA subsurface samples. In contrast, q_fast_ did not display significant differences between sampling regions. Notable regional differences in NPQ dynamics appeared to be related to variability in mean daily irradiance exposure (Table 1). Across the three datasets, mean daily irradiance displayed a positive correlation with q_int_ (*r* = 0.39, *p* < < 0.01), and negative correlations with slow q_slow_ (*r* = −0.45, *p* < < 0.01) and $\tau_{qN}$ (*r* = −0.46, *p* < < 0.01). This result provides evidence that light acclimation status influences NPQ relaxation dynamics, with rapidly-relaxing components of NPQ preferentially upregulated under higher light conditions.

Although the amplitudes of q_fast_, q_int_ and q_slow_ varied between samples, relaxation lifetimes ( $\tau_{fast},\tau_{\mathrm{int}},\tau_{slow}$ ) for each component were remarkably similar (Table 1), supporting their interpretation as reflecting distinct mechanisms. Consistent with previous observations (Malnoë, 2018), $\tau_{fast}$ was ~30s, while $\tau_{\mathrm{int}}$ was around 5 min, and $\tau_{slow}$ was several hours.

Previous studies of NPQ kinetics have attributed fast relaxation quenching (lifetimes ranging from ~10 to 100 s) to rapid energy-dependent quenching (qE) associated PsbS de-protonation. Intermediate quenching (life times around 10 min; Schansker et al., 2006; Roháček, 2010) have been interpreted as state-transition related quenching (qT), while and slow quenching relaxing (over several hours) has been attributed to long-lived photoinhibition (qI; Nilkens et al., 2010; Roháček, 2010). We observed that intermediate quenching was the most significant contributor to qN_0_ across all samples, with relaxation life times faster than LHC de-phosphorylation rates required to reverse state transitions (McCormac et al., 1994), but within range of zeaxanthin epoxidation rates (Nilkens et al., 2010). We thus postulate that the intermediate component of NPQ relaxation, commonly attributed to qT in other studies, may be associated with xanthophyll cycling dynamics (qZ) in our samples, rather than with state transitions. This idea is supported by the significantly higher PP:PS ratios in surface CAA samples (with high q_int), relative to subsurface samples. Further, qT pathways are not known to exist in diatoms (Lavaud, 2007), which were prevalent in our study regions, particularly in southern Baffin Bay (See section 4.3.2, and Supplementary material S2). Diatoms have instead been shown to exhibit strong xanthophyll-regulated quenching (Blommaert et al., 2021), consistent with our observations.

### 4.2 Correlation between $\sigma_{PSII}$ and F_V_/F_M_ during NPQ relaxation and implications for ETR estimates

**Figure 3:**
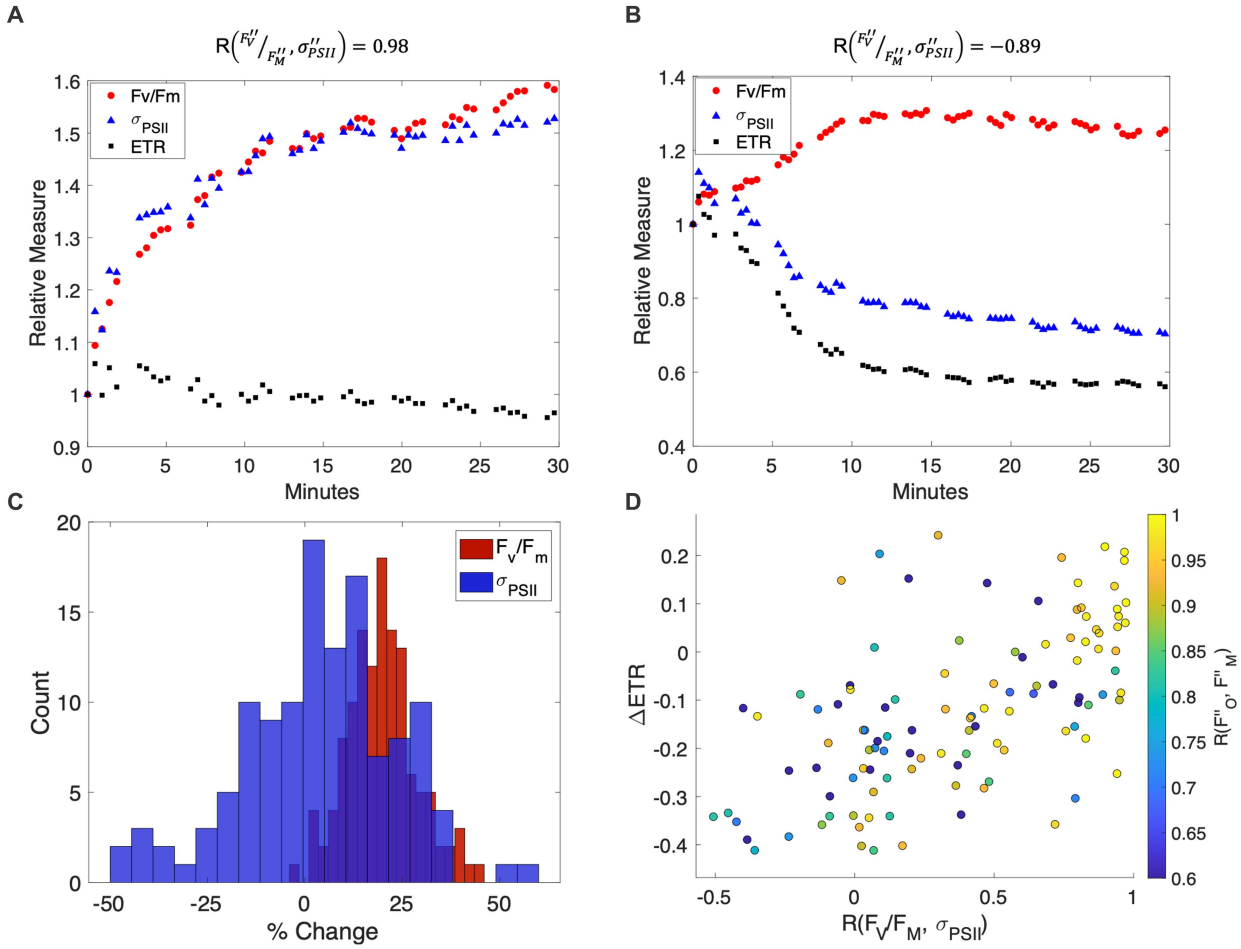
**(A,B)** Representative time-series of $\sigma \prime \prime_{\mathrm{PSII}}$ and F ″ _V_/F ″ _M_ measurements during NPQ relaxation, showing strong coupling **(A)** and decoupling **(B)** of these variables following low light exposure. The percent change of $\sigma \prime \prime_{\mathrm{PSII}}$ (blue triangles), F ″ _V_/F ″ _M_ (red circles), and ETR (black squares) are plotted with respect to NPQ relaxation time in minutes, with values normalized to 1 at the beginning of the low light measurement period. **(C)** Distribution of F ″ _V_/F ″ _M_ (blue) and $\sigma \prime \prime_{\mathrm{PSII}}$ (orange) changes during NPQ relaxation experiments. Changes in F ″ _V_/F ″ _M_ were always positive, whereas both positive and negative changes in $\sigma \prime \prime_{\mathrm{PSII}}$ were observed. **(D)** The effect of $\sigma \prime \prime_{\mathrm{PSII}}$ and F ″ _V_/F ″ _M_ decoupling on derived ETR estimates. Samples with strongly correlated $\sigma \prime \prime_{\mathrm{PSII}}$ and F ″ _V_/F ″ _M_ also displayed proportional $F_{M}^{\prime \prime }$ and $F_{O}^{\prime \prime }$ responses (correlation coefficients shown on the colorbar) and displayed little change in ETR estimates during the time-course of NPQ.

As expected, NPQ relaxation led to a consistent increase in F ″ _V_/F ″ _M_ following the removal of high light (Figure 3C). In contrast, there was much greater variability in $\sigma \prime \prime_{PSII}$ responses to NPQ relaxation, with instances of both increasing and decreasing $\sigma \prime \prime_{PSII}$ over the time-course of low light exposure. As a result, changes in F ″ _V_/F ″ _M_ and $\sigma \prime \prime_{PSII}$ were sometimes uncoupled during NPQ relaxation, with correlation coefficients ranging from 0.98 to −0.89 (e.g., Figures 3A,B) across different measurement locations. Divergence between F ″ _V_/F ″ _M_ and $\sigma \prime \prime_{PSII}$ exerted a direct effect on derived ETR estimates during NPQ relaxation. As hypothesized, samples with synchronous changes in F ″ _V_/F ″ _M_ and $\sigma \prime \prime_{PSII}$ displayed minimal changes in ETR estimates (Eq. 5) over the time course of the experiment (Figures 3A,D). In contrast, ETR decreased by as much as 40% in samples where F ″ _V_/F ″ _M_ and $\sigma \prime \prime_{PSII}$ showed significant uncoupling (Figures 3B,D). These results challenge the notion that ETR estimates are unaffected by NPQ relaxation time due to synchronous F ″ _V_/F ″ _M_ and $\sigma \prime \prime_{PSII}$ responses.

The expectation of equal NPQ effects on $\sigma_{PSII}$ and F_V_/F_M_ is based on the definition of $\sigma_{PSII}$ as the product of F_V_/F_M_ and the absorption coefficient of PSII photochemistry, $a_{PSII}$ . In turn, $a_{PSII}$ , is determined by the ratio of the absorption coefficient of light harvesting complexes ( $a_{LHC}$ ) and the concentration of functional PSII reaction centers (Silsbe et al., 2015). During ST-ChlF protocols, $a_{PSII}$ is typically assumed to be constant over the relatively short time-course of measurements. In practice, however, a decrease in $\sigma_{PSII}$ during NPQ relaxation could be explained by increasing numbers of functional PSII reaction centers, as cells recover from photoinhibition, such that $a_{LHC}$ is “diluted” across more PSII centers.

We examined the potential role of photoinhibition in the NPQ signal, based on the correlation between $F_{o}^{\prime \prime }$ and $F_{M}^{\prime \prime }$ during NPQ relaxation curves. Regulated NPQ (qE and qZ) is expected to cause proportional quenching of F_o_ and F_M_, while photoinhibition leads to increased F_o_ relative to F_M_ (Gilmore et al., 1996; Müller et al., 2001). Our analysis showed that the relationship between $\sigma \prime \prime_{PSII}$ and NPQ was related to underlying patterns in $F_{o}^{\prime \prime }$ and $F_{M}^{\prime \prime }$ . Across all samples, $F_{o}^{\prime \prime }$ and $F_{M}^{\prime \prime }$ increased during NPQ relaxation periods, but subtle differences in $F_{o}^{\prime \prime }$ and $F_{M}^{\prime \prime }$ recovery kinetics led to some variability in the relationship between the two terms, with correlation coefficients ranging from 0.55 to >0.99 (Figure 3D). Out of 125 NPQ relaxation experiments, 39 samples displayed perfectly synchronized $F_{o}^{\prime \prime }$ and $F_{m}^{\prime \prime }$ responses (*r* > = 0.99). Among these samples, $\sigma \prime \prime_{PSII}$ increased over the time-course of NPQ relaxation and showed a strong negative correlation with NPQ (*r* = −0.87). These samples also displayed tightly coupled $\sigma \prime \prime_{PSII}$ and F ″ _V_/F ″ _M_ responses (*r =* 0.80), near constant ETR estimates during the NPQ relaxation time course (*r* = −0.02), and only a small contribution of long-lived photoinhibitory quenching (q_slow_) to the overall qN signal (q_slow_ = 0.02 ± 0.01). In contrast, samples with less synchronized $F_{o}^{\prime \prime }$ and $F_{M}^{\prime \prime }$ responses (*r* < = 0.90) displayed higher degrees of q_slow_ (0.08 ± 0.01), and weaker relationships between $\sigma \prime \prime_{PSII}$ and F ″ _V_/F ″ _M_ (*r* = 0.20). In these latter samples, ETR estimates were more sensitive to dark relaxation, decreasing in response to relaxation life time (*r* = −0.35). These results suggest that the assumption of constant $a_{PSII}$ may be violated due to plasticity in the concentration of functional RCIIs under conditions of initial photoinhibition followed by recovery. It follows from this that choice of NPQ relaxation protocol will have a particularly strong influence on ETR results in samples exposed to potential photoinhibitory light regimes.

### 4.3 Environmental and taxonomic controls on NPQ relaxation kinetics and ETR estimates

The considerable variability we observed in NPQ relaxation kinetics reflects differences in the photo-acclimation state of phytoplankton assemblages across our study region. This photophysiological variability is, in turn, driven by hydrographic and taxonomic properties associated with spatial and temporal (summer vs. fall) differences between samples. To minimize confounding seasonal effects, we conducted separate analysis of environmental and taxonomic effects on NPQ relaxation dynamics and ETR for the CAA and Svalbard datasets.

#### 4.3.1 Svalbard coastal oceanography and photophysiology

**Figure 4:**
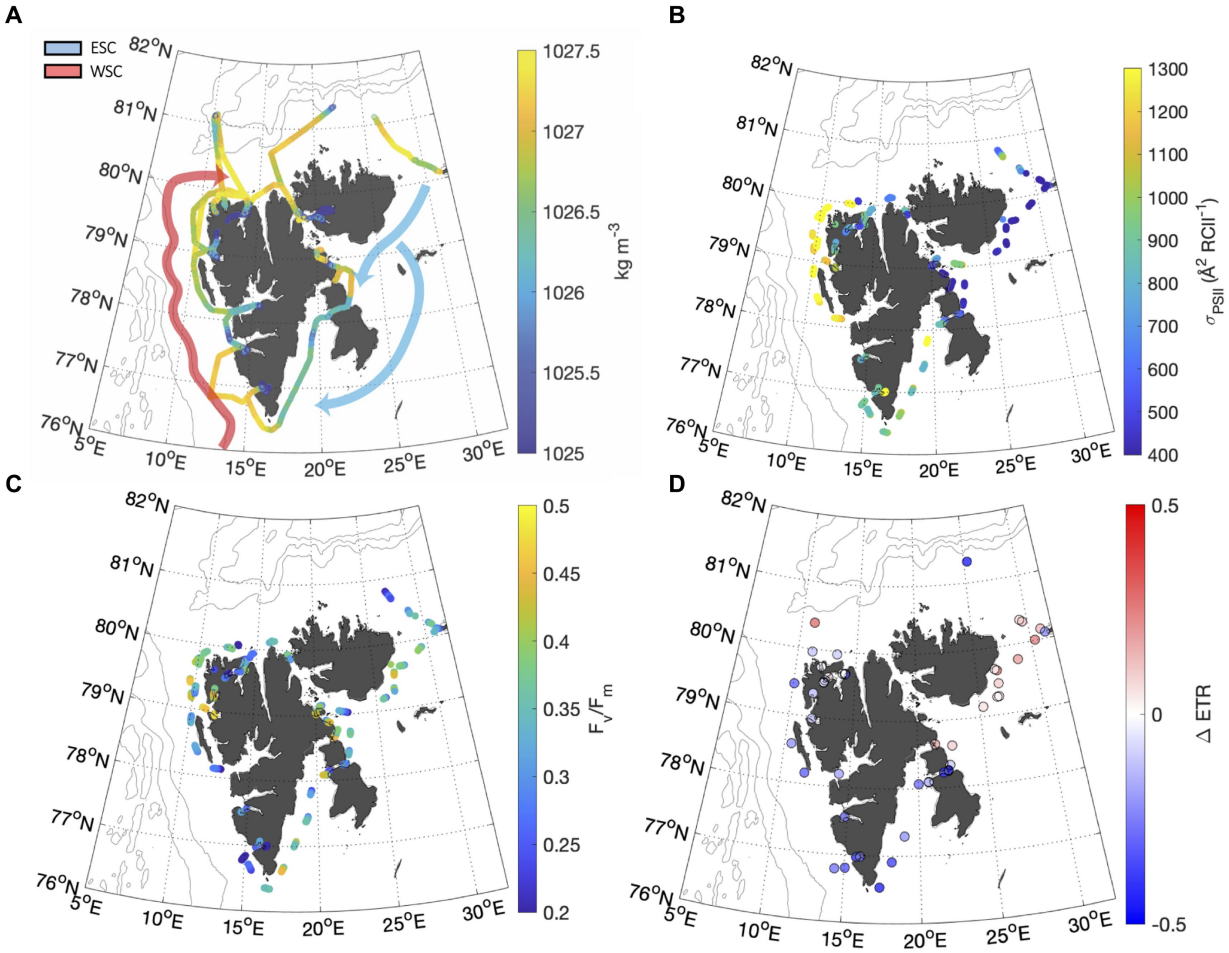
**(A)** Distribution of seawater density along the ship track calculated from salinity and temperature measurements (Millero and Poisson, 1981) illustrate the hydrographic difference between the West and East Spitsbergen Current (WSC and ESC, respectively). The WSC was defined as waters saltier than 35 psu and warmer than 6°C (Aagaard et al., 1987). Fjords were classified as water with a salinity <33 psu. All intermediate water was classified as ESC. Gaps in thermosalinograph (TSG) data occurred during sampling in heavily ice-covered waters. Approximate paths of the WSC (red) and ESC (white) are adapted from Vihtakari et al. (2018). Panels **(B,C)** show continuous underway $\sigma_{PSII}$ and F_V_/F_M_ measurements. Panel **(D)** shows the fractional change in ETR recorded throughout NPQ relaxation experiments.

The Svalbard Archipelago is situated on the eastern boundary of Fram Strait, where the bulk of water mass transfer occurs between the Arctic and Atlantic basins (Walczowski, 2014). Off the west coast of Svalbard, the West Spitsbergen Current (WSC) delivers warm, saline Atlantic water north to the Arctic Basin, while off the east coast, the East Spitsbergen Current (ESC) carries cold, fresher Arctic water south. These currents mix with glacial melt water in the many fjords penetrating the Svalbard coastline. Due to the distinct hydrographic features of these different water masses, we were able to use underway temperature and salinity measurements to divide data into WSC, ESC and Fjord subregions (Figure 4A).

Photophysiological properties varied with hydrographic variables such as temperature, salinity and PAR (Table 2), leading to significant spatial heterogeneity along our cruise track (Figures 4B-D). Most notably, $\sigma_{PSII}$ varied significantly from east to west, with a mean value of 1,309 ± 7.15 Å^2^ RCII^−1^ in the WSC, compared to 745 ± 8 and 696 ± 7 Å^2^ RCII^−1^ for fjords and the ESC, respectively (Figure 4B). In contrast, there were no significant differences in F_V_/F_M_ between any of the subregions, with mean values falling within 0.33 to 0.36 for the three subregions (Figure 4C). Variability in $\sigma_{PSII}$ had a significant effect on NPQ relaxation kinetics and ETR behavior across the subregions (Table 2). In ESC-influenced waters with lower $\sigma_{PSII}$ , ETR was relatively stable during NPQ relaxation experiments, with a mean percent change of −2.5 ± 5.0%. By comparison, ETR estimates in WSC and fjord waters were more sensitive to NPQ relaxation, with ΔETR of −10.1 ± 7.0%. and − 24.1 ± 3.6%, respectively. Differences in ΔETR were significant between Fjord and ESC populations (*p* << 0.01).

**Table 2:** Spearman rank correlation coefficients between temperature (°C), salinity (psu), surface PAR ( μ mol photons m^−2^ s^−1^) and photo-physiological parameters.

	$\sigma_{PSII}$	F_V_/F_M_	qN	$\tau_{qN}$	q_fast_	q_int_	q_slow_	Δ ETR
Temp	0.73**	−0.55*	0.37	0.10	0.30*	−0.33*	0.06	−0.07
Sal	0.59*	0.16	0.71**	−0.23	−0.14	0.13	0.09	0.34*
PAR	0.37	−0.57**	−0.17	−0.10	0.59**	−0.55*	−0.35	0.28

Values of *p* < 0.05 are indicated with * and values of *p* << 0.01 with **. Sample number, n, is 58 in all cases.

Variability in phytoplankton taxonomy may partially explain the spatial differences in photo-physiology around Svalbard. Different phytoplankton taxa have distinct light harvesting pigment compositions, and the functional PSII absorption area, $\sigma_{PSII}$ , also shows taxon-specific variability (Suggett et al., 2009; Gorbunov et al., 2020). Although F_V_/F_M_ also varies with species composition, it appears to be more strongly affected by nutrient concentrations, and is thus widely used as an indicator of environmental conditions (Ryan-Keogh et al., 2013; Ko et al., 2020). The significant differences we observed in $\sigma_{PSII}$ but not in F_V_/F_M_ suggest that variability between subregions of the Svalbard Archipelago are due to changes in taxonomic composition, rather than direct environmental effects. Although we lack direct estimates of phytoplankton taxonomy for the Svalbard region (due to limited opportunities for discrete sampling), previous studies in this area have noted differences in phytoplankton composition between ESC and WSC influenced waters. Kilias et al. (2014) observed a high percentage of haptophytes in WSC study sites, with chlorophytes more abundant at a study site affected by the ESC. The differences in $\sigma_{PSII}$ between the ESC and WSC reported here agree well with expected values for haptophytes and chlorophytes. Laboratory monocultures of green algae yielded 450 nm-specific measurements of $\sigma_{PSII}$ nearing 600 Å^2^ RCII^−1^, while haptophytes were closer to 1,000 Å^2^ RCII^−1^ (Gorbunov et al., 2020). Without ancillary data, we cannot unequivocally determine whether observed differences in $\sigma_{PSII}$ , and ultimately ETR behavior, are driven by differences in nutrient availability or in phytoplankton taxonomy for the Svalbard samples. As discussed below, such analysis is, however, possible for the CAA samples.

#### 4.3.2 Canadian Arctic oceanography and photophysiology

**Figure 5:**
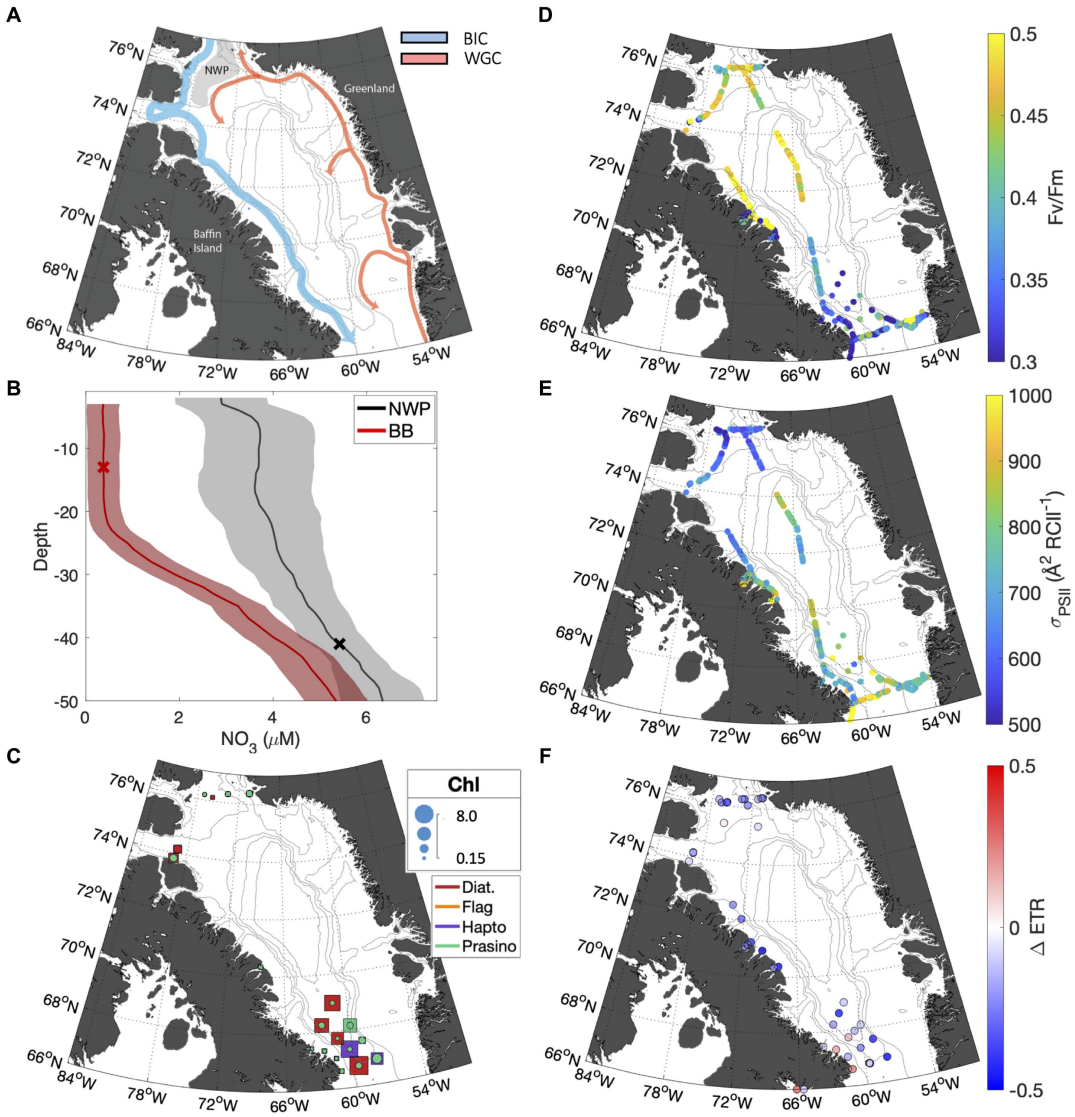
**(A)** Map of Baffin Bay showing bathymetry and surface currents. The southbound Baffin Island Current (BIC) is shown in blue, and the northbound West Greenland Current (WGC) in red. The gray shaded area highlights the NWP. **(B)** Mean nitrate depth profiles are displayed for Baffin Bay stations (red), and NWP stations (black). The standard error is indicated by the shaded area. The mean mixed layer depth for both regions is indicated by X marker. **(C)** Taxonomic distribution of phytoplankton assemblages; colors indicate the dominant taxa group for the subsurface (squares) and surface (circles), while marker size indicates the total chlorophyll concentration. The F_V_/F_M_ and $\sigma_{PSII}$ for underway surface samples are shown in subplots **(D,E)**, respectively. **(F)** The fractional change in ETR over the NPQ relaxation time (30 min) for surface samples.

Sampling in the Canadian Arctic (CAA) took place in the late summer to early fall (Sept 23 – Oct 15, 2022) in Baffin Bay, primarily in waters influenced by the Baffin Island current (Figure 5A). This water mass transports Pacific-derived Arctic water exiting Nares Strait and Lancaster Sound south to the Labrador Sea along the east coast of Baffin Island (Münchow et al., 2015). In the north, the cruise track intersected the North Water Polynya (NWP), where southbound Arctic water mixes with warmer, saltier Atlantic water carried by the West Greenland Current. The polynya, referred to by local communities as Pikialasorsuaq or "great upwelling,” has long been recognized as a productivity hotspot (Ribeiro et al., 2021). As discussed below, biogeochemical and photophysiological differences were apparent between the different oceanographic settings of the NWP and Southern Baffin Bay.

Depth profiles of seawater density within the NWP (stations with latitude >76^o^N) confirmed enhanced vertical mixing in the NWP. The average mixed layer (ML) depth in the NWP was 41.5 ± 16.9 m. By comparison, stratification was stronger in Southern Baffin Bay, where the ML was 20.4 ± 2.22 m (Figure 5B). As expected, differences in stratification intensity between Baffin Bay and the NWP were associated with differences in surface water nutrient concentrations; [NO_3_] was elevated in NWP surface waters compared to Baffin stations, with mean concentrations of 3.46 ±1.11 and 0.37 ± 0.34 *µ*M, respectively (Figure 5B). The higher nutrient availability in the NWP surface waters was associated with elevated F_V_/F_M_ values (Table 3; Figure 5D). However, the highest biomass was observed in sub-surface waters of central Southern Baffin Bay (Figure 5C), in a deeper chlorophyll maximum where [Chl] peaked at 8.1 mg m^−3^ and the associated F_V_/F_M_ was 0.48. This elevated biomass may have contributed to the depleted nitrate values in Baffin Bay surface and subsurface waters.

**Table 3:** Spearman rank correlation coefficients between temperature (°C), salinity (psu), PAR ( μ mol photons m^−2^ s^−1^), PP:PS (dimensionless), NO_3_ (µM), and Chl (µg L^−1^) and photo-physiological parameters in the Canadian Arctic Archipelago.

	$\sigma_{PSII}$	F_V_/F_M_	qN	$\tau_{qN}$	q_fast_	q_int_	q_slow_	Δ ETR	n
Surface									
Temp	0.26	−0.29	−0.42*	0.08	0.11	−0.19	0.16	0.22	48
Sal	−0.53**	0.19	0.15	0.18	0.26	−0.23	0.36	0.47*	48
PAR	0.19	−0.19	−0.17	−0.11	−0.06	−0.07	−0.05	0.09	48
PP:PS	0.58**	−0.09	−0.38	−0.15	−0.43*	0.39*	−0.11	−0.18	19
NO_3_	−0.34	0.51*	−0.05	−0.09	−0.12	0.16	0.11	−0.19	19
Chl	−0.06	0.30*	0.24	0.32	−0.23	0.27	0.02	−0.02	19
Sub-surface									
PAR	0.26	−0.43	−0.19	−0.38	−0.01	0.17	−0.25	−0.22	19
PP:PS	−0.45*	0.62**	0.00	−0.15	−0.45*	0.17	0.22	−0.58*	19
NO_3_	−0.14	0.37	−0.11	0.00	−0.11	0.31	−0.11	−0.17	19
Chl	0.23	−0.42	0.37	0.04	0.26	0.03	−0.20	0.16	19

Values of *p* < 0.05 are indicated with *. Values of *p* << 0.01 are **. The number of samples, n, is shown in the right-hand column.

Across our CAA sampling stations, the ratio of photoprotective to photosynthetic pigments (PP:PS) was the strongest predictor of NPQ relaxation dynamics (Table 3), reflecting the influence of light-acclimation state on NPQ responses. PP:PS also displayed strong correlations with $\sigma_{PSII}$ , albeit with an opposite relationship in surface waters (positive correlation) and subsurface waters (negative correlation). The positive correlation between PP:PS and $\sigma_{PSII}$ in surface samples is not immediately intuitive, as photoprotective pigments are not excitonically coupled to RCII. However, as $\sigma_{PSII}$ is dependent on the ratio of $a_{LHC}$ to [RCII], samples with relatively few photosynthetic pigments are expected to have limited [RCII], leading to elevated $\sigma_{PSII}$ (see section 4.2). In turn, $\sigma_{PSII}$ was the greatest determinant of ETR behavior during NPQ relaxation experiments among surface samples. Surface $\sigma_{PSII}$ was negatively correlated with ΔETR ( ρ = −0.75, *p* < <0.01), such that samples with lower $\sigma_{PSII}$ displayed only small changes in ETR during NPQ relaxation experiments. In contrast, samples with higher $\sigma_{PSII}$ values demonstrated large decreases in ETR estimates as a function of NPQ relaxation time. The relationship between $\sigma_{PSII}$ and ΔETR observed here is similar to that reported for Svalbard above.

In contrast to surface waters, there was no statistically significant correlation between $\sigma_{PSII}$ and ΔETR in subsurface samples ( ρ = 0.34, *p* > 0.05). We did, however, observe a significant relationship between PP:PS and ΔETR. Samples with lower concentrations of photo-protective pigments (i.e., lower PP:PS ratios) exhibited larger decreases in ETR estimates during the time-course of NPQ relaxation (Table 3). This result can be understood in the context of cellular photo-protective capacity. Cells with lower concentrations of photoprotective pigments would be more prone to long-lived photoinhibition during high light treatments (Choudhury and Behera, 2001), which could explain the observed decreases in $\sigma \prime \prime_{PSII}$ (and thus ETR) during NPQ relaxation (See section 4.2). These results imply that light acclimation state and species composition, plays an important role in determining the stability of ETR during NPQ relaxation experiments.

Beyond the use of PP:PS and $\sigma_{PSII}$ as indicators of cellular photo-protective capacity, these light harvesting properties provide some taxonomic explanation for the observed patterns in ΔETR. Diatom-dominated stations exhibited the lowest $\sigma_{PSII}$ values and ΔETR closest to zero, whereas higher $\sigma_{PSII}$ were observed at stations dominated by picoplankton, and where absolute magnitudes of ΔETR were greater (Supplementary material S2). This result suggests that photosynthetic rates estimates are less sensitive to NPQ relaxation effects in diatom-dominated assemblages, as compared to picoplankton dominated communities. Assumptions of proportional NPQ effects on $\sigma_{PSII}$ and F_V_/F_M_, and thus NPQ effects on photosynthetic rate estimates, were developed in early fluorescence studies conducted on eukaryotic species (Gorbunov et al., 2001; Suggett et al., 2010). However, previous studies have shown these assumptions do not hold in prokaryotes (Xu et al., 2018), and our results provide field-based evidence that they are also violated in marine environments with taxonomically-mixed phytoplankton assemblages.

## 5 Conclusion

The purpose of this study was to characterize the effects of natural oceanographic variability on NPQ relaxation kinetics, and the subsequent effects of NPQ relaxation on ETR estimates. Contrary to the assumption that NPQ relaxation time should have no influence on ETR estimates, we observed divergent F_V_/F_M_ and $\sigma_{PSII}$ responses during NPQ relaxation experiments, leading to significant NPQ-dependent variability in ETR estimates. We thus conclude that NPQ relaxation time is, indeed, an important consideration for reliable ETR estimates. On average, we found that 20 min of low light exposure was sufficient to relax short-lived quenching mechanisms, while 45 min was sufficient for samples acclimated to low light environments. These results have important implications for the application of FRRF in underway surveys of photochemistry. Distinct NPQ dynamics occur across varying light environments and phytoplankton taxonomic composition, and can vary strongly across different hydrographic subregions. We recommend researchers apply their own NPQ relaxation experiments at the onset of a new field campaign to determine the most appropriate NPQ relaxation period for their study region. With an increasing dataset of NPQ dynamics across a range of oceanographic regions, it may be possible to build empirical models defining appropriate protocols for NPQ relaxation for field-based studies.

## Data availability statement

The datasets presented in this study can be found in online repositories. The names of the repository/repositories and accession number(s) can be found in the article/Supplementary material.

## Author contributions

YS: Data curation, Formal analysis, Writing – original draft. DC: Investigation, Writing – review & editing. SP: Formal analysis, Writing – review & editing. PT: Funding acquisition, Resources, Supervision, Writing – review & editing.

## References

[ref1] Aagaard K. Foldvik A. Hillman S. R. (1987). The West Spitsbergen current: disposition and water mass transformation. J. Geophys. Res. Oceans 92, 3778–3784. doi: 10.1029/JC092iC04p03778

[ref2] Alderkamp A. C. de Baar H. J. W. Visser R. J. W. Arrigo K. R. (2010). Can photoinhibition control phytoplankton abundance in deeply mixed water columns of the Southern Ocean?’. Limnol. Oceanogr. 55, 1248–1264. doi: 10.4319/lo.2010.55.3.1248

[ref3] Alderkamp A. C. Mills M. M. van Dijken G. Arrigo K. R. (2013). Photoacclimation and non-photochemical quenching under in situ irradiance in natural phytoplankton assemblages from the Amundsen Sea, Antarctica’. Mar. Ecol. Prog. Ser. 475, 15–34. doi: 10.3354/meps10097

[ref4] Ardyna M. Babin M. Gosselin M. Devred E. Rainville L. A. (2014). Recent Arctic Ocean sea ice loss triggers novel fall phytoplankton blooms. Geophys. Res. Lett. 41, 6207–6212. doi: 10.1002/2014GL061047.Received

[ref5] Blommaert L. Chafai L. Bailleul B. (2021). The fine-tuning of NPQ in diatoms relies on the regulation of both xanthophyll cycle enzymes. Scientific Reports. Nature Publishing Group UK 11, 12750–12716. doi: 10.1038/s41598-021-91483-x, PMID: 34140542 PMC8211711

[ref6] Campbell D. A. Serôdio J. (2020). “Photoinhibition of photosystem II in phytoplankton: processes and patterns” in Photosynthesis in algae: Biochemical and physiological mechanisms. eds. Larkum A. W. D. Grossman A. R. Raven J. A. (Cham: Springer International Publishing), 329–365.

[ref7] Choudhury N. K. Behera R. K. (2001). Photoinhibition of photosynthesis: role of carotenoids in Photoprotection of chloroplast constituents. Photosynthetica 39, 481–488. doi: 10.1023/A:1015647708360

[ref8] Chukhutsina V. U. Büchel C. Van Amerongen H. (2014). Disentangling two non-photochemical quenching processes in *Cyclotella meneghiniana* by spectrally-resolved picosecond fluorescence at 77 K. Biochimica et Biophysica Acta - Bioenergetics. Elsevier B.V. 1837, 899–907. doi: 10.1016/j.bbabio.2014.02.021, PMID: 24582663

[ref9001] Coupel P. Matsuoka A. Ruiz-Pino D. Gosselin M. Marie D. Tremblay J.-É.. (2015). ‘Pigment signatures of phytoplankton communities in the Beaufort Sea’, Biogeosciences 12, 991–1006. doi: 10.5194/bg-12-991-2015

[ref9] Croteau D. Guérin S. Bruyant F. Ferland J. Campbell D. A. Babin M.. (2021). Contrasting nonphotochemical quenching patterns under high light and darkness aligns with light niche occupancy in Arctic diatoms. Limnol. Oceanogr. 66, S231–S245. doi: 10.1002/lno.11587

[ref10] Fernández-Marín B. Roach T. Verhoeven A. García-Plazaola J. I. (2021). Shedding light on the dark side of xanthophyll cycles. New Phytol. 230, 1336–1344. doi: 10.1111/nph.17191, PMID: 33452715

[ref9002] Gilmore A. M. Hazlett T. L. Debrunner P. G., (1996). Comparative time-resolved photosystem II chlorophyll a fluorescence analyses reveal distinctive differences between photoinhibitory reaction center damage and Xanthophyll cycle-dependent energy dissipation. Photochem. Photobiol. 64, 552–563. doi: 10.1111/j.1751-1097.1996.tb03105.x 8806231

[ref11] Gorbunov M. Y. Kolber Z. S. Lesser M. P. Falkowski P. G. (2001). Photosynthesis and photoprotection in symbiotic corals. Limnol. Oceanogr. 46, 75–85. doi: 10.4319/lo.2001.46.1.0075

[ref12] Gorbunov M. Y. Kuzminov F. I. Fadeev V. V. Kim J. D. Falkowski P. G. (2011). A kinetic model of non-photochemical quenching in cyanobacteria. Biochimica et Biophysica Acta (BBA)-Bioenergetics 1807, 1591–1599. doi: 10.1016/j.bbabio.2011.08.009, PMID: 21907180

[ref13] Gorbunov M. Y. Shirsin E. Nikonova E. Fadeev V. V. Falkowski P. G. (2020). A multi-spectral fluorescence induction and relaxation (fire) technique for physiological and taxonomic analysis of phytoplankton communities. Mar. Ecol. Prog. Ser. 644, 1–13. doi: 10.3354/meps13358

[ref14] Goss R. Lepetit B. (2015). Biodiversity of NPQ. J. Plant Physiol. 172, 13–32. doi: 10.1016/j.jplph.2014.03.004 24854581

[ref9003] Higgins H. Wright S. Schlüter L. (2011). “Quantitative interpretation of chemotaxonomic pigment data.” Phytoplankton Pigments: Characterization, Chemotaxonomy and Applications in Oceanography. Eds. Roy S. Llewellyn C. Egeland E. Johnsen G. (Cambridge Environmental Chemistry Series). Cambridge:Cambridge University Press. 257–313

[ref15] Hughes D. J. Varkey D. Doblin M. A. Ingleton T. Mcinnes A. Ralph P. J.. (2018). Impact of nitrogen availability upon the electron requirement for carbon fixation in Australian coastal phytoplankton communities. Limnol. Oceanogr. 63, 1891–1910. doi: 10.1002/lno.10814

[ref9004] Jeffrey S. W. Mantoura R. F. C. Wright S. W. (1997). Phytoplankton pigments in oceanography: guidelines to modern methods. Paris: UNESCO Pub.

[ref9005] Kilias E. S. Nöthig E.-M. Wolf C. Metfies K. (2014). Picoeukaryote Plankton Composition off West Spitsbergen at the Entrance to the Arctic Ocean. J. Eukaryot. Microbiol. 61, 569–579. doi: 10.1111/jeu.12134 24996010

[ref16] Ko E. Gorbunov M. Y. Jung J. Joo H. M. Lee Y. Cho K. H.. (2020). Effects of nitrogen limitation on phytoplankton physiology in the Western Arctic Ocean in summer. J. Geophys. Res. Oceans 125, 1–19. doi: 10.1029/2020JC016501

[ref17] Kolber Z. S. Prášil O. Falkowski P. G. (1998). Measurements of variable chlorophyll fluorescence using fast repetition rate techniques: defining methodology and experimental protocols. Biochim. Biophys. Acta Bioenerg. 1367, 88–106. doi: 10.1016/S0005-2728(98)00135-2, PMID: 9784616

[ref18] Kromkamp J. C. Forster R. M. (2003). The use of variable fluorescence measurements in aquatic ecosystems: differences between multiple and single turnover measuring protocols and suggested terminology. Eur. J. Phycol. 38, 103–112. doi: 10.1080/0967026031000094094

[ref19] Lacour T. Babin M. Lavaud J. (2020). Diversity in xanthophyll cycle pigments content and related nonphotochemical quenching (NPQ) among microalgae: implications for growth strategy and ecology. J. Phycol. 56, 245–263. doi: 10.1111/jpy.12944 31674660

[ref20] Lacour T. Larivière J. Ferland J. Bruyant F. Lavaud J. Babin M. (2018). The role of sustained Photoprotective non-photochemical quenching in low temperature and high Light acclimation in the bloom-forming Arctic diatom Thalassiosira gravida. Front. Mar. Sci. 5. doi: 10.3389/fmars.2018.00354

[ref21] Lavaud J. (2007). Fast regulation of photosynthesis in diatoms: mechanisms, evolution and ecophysiology. Functional Plant Sci. Biotechonol. 1, 267–287.

[ref22] Lewis K. M. Arntsen A. E. Coupel P. Joy-Warren H. Lowry K. E. Matsuoka A.. (2019). Photoacclimation of Arctic Ocean phytoplankton to shifting light and nutrient limitation. Limnol. Oceanogr. 64, 284–301. doi: 10.1002/lno.11039

[ref23] Lewis K. M. Van Dijken G. L. Arrigo K. R. (2020). Changes in phytoplankton concentration now drive increased Arctic Ocean primary production. Science 369, 198–202. doi: 10.1126/science.aay8380 32647002

[ref24] Li Z. Li W. Zhang Y. Hu Y. Sheward R. Irwin A. J.. (2021). Dynamic Photophysiological stress response of a model diatom to ten environmental stresses. J. Phycol. 57, 484–495. doi: 10.1111/jpy.13072 32945529

[ref25] Long S. P. Taylor S. H. Burgess S. J. Carmo-Silva E. Lawson T. de Souza A. P.. (2022). Into the shadows and Back into sunlight: photosynthesis in fluctuating Light. Annu. Rev. Plant Biol. 73, 617–648. doi: 10.1146/annurev-arplant-070221-024745, PMID: 35595290

[ref26] Malnoë A. (2018). Photoinhibition or photoprotection of photosynthesis? Update on the (newly termed) sustained quenching component qH. Environ. Exp. Bot. 154, 123–133. doi: 10.1016/j.envexpbot.2018.05.005

[ref27] Matrai P. A. Olson E. Suttles S. Hill V. Codispoti L. A. Light B.. (2013). Synthesis of primary production in the Arctic Ocean: I. Surface waters, 1954-2007. Prog. Oceanogr. 110, 93–106. doi: 10.1016/j.pocean.2012.11.004

[ref28] McCormac D. J. Bruce D. Greenberg B. M. (1994). State transitions, light-harvesting antenna phosphorylation and light-harvesting antenna migration in vivo in the higher plant *Spirodela oligorrhiza*. Biochimica et Biophysica Acta (BBA)-Bioenergetics 1187, 301–312. doi: 10.1016/0005-2728(94)90004-3

[ref29] Millero F. J. Poisson A. (1981). ‘International one-atmosphere equation of state of seawater’, Deep Sea research part A. Oceanographic Res. Papers 28, 625–629. doi: 10.1016/0198-0149(81)90122-9

[ref30] Müller P. Li X. P. Niyogi K. K. (2001). Non-photochemical quenching. A response to excess light energy. Plant Physiol. 125, 1558–1566. doi: 10.1104/pp.125.4.1558, PMID: 11299337 PMC1539381

[ref31] Münchow A. Falkner K. K. Melling H. (2015). Baffin Island and West Greenland current systems in northern Baffin Bay. Prog. Oceanogr. 132, 305–317. doi: 10.1016/j.pocean.2014.04.001

[ref32] Nilkens M. Kress E. Lambrev P. Miloslavina Y. Müller M. Holzwarth A. R.. (2010). Identification of a slowly inducible zeaxanthin-dependent component of non-photochemical quenching of chlorophyll fluorescence generated under steady-state conditions in Arabidopsis. Biochimica et Biophysica Acta (BBA) - Bioenergetics 1797, 466–475. doi: 10.1016/j.bbabio.2010.01.001, PMID: 20067757

[ref33] Noh Y. Lee W.-S. (2008). Mixed and mixing layer depths simulated by an OGCM. J. Oceanogr. 64, 217–225. doi: 10.1007/s10872-008-0017-1

[ref34] Owens T. G. (1986). Light-harvesting function in the diatom *Phaeodactylum tricornutum*. Plant Physiol. 80, 739–746. doi: 10.1104/pp.80.3.739, PMID: 16664695 PMC1075193

[ref35] Ribeiro S. Limoges A. Massé G. Johansen K. L. Colgan W. Weckström K.. (2021). Vulnerability of the north water ecosystem to climate change. Nat. Commun. 12:4475. doi: 10.1038/s41467-021-24742-0, PMID: 34294719 PMC8298575

[ref36] Roháček K. (2010). Method for resolution and quantification of components of the non-photochemical quenching (qN). Photosynth. Res. 105, 101–113. doi: 10.1007/s11120-010-9564-6, PMID: 20535559

[ref37] Roháček K. Bertrand M. Moreau B. Jacquette B. Caplat C. Morant-Manceau A.. (2014). Relaxation of the non-photochemical chlorophyll fluorescence quenching in diatoms: kinetics, components and mechanisms. Philos. Trans. Royal Society B: Biolog. Sci. 369:20130241. doi: 10.1098/rstb.2013.0241 PMC3949399 24591721

[ref38] Ryan-Keogh T. J. Macey A. I. Nielsdóttir M. C. Lucas M. I. Steigenberger S. S. Stinchcombe M. C.. (2013). Spatial and temporal development of phytoplankton iron stress in relation to bloom dynamics in the high-latitude North Atlantic Ocean. Limnol. Oceanogr. 58, 533–545. doi: 10.4319/lo.2013.58.2.0533

[ref39] Ryan-keogh T. J. Thomalla S. J. (2020). Deriving a Proxy for Iron Limitation From Chlorophyll Fluorescence on Buoyancy Gliders’. Front. Mar. Sci. 7, 1–13. doi: 10.3389/fmars.2020.0027532802822

[ref40] Schallenberg C. Strzepek R. F. Schuback N. Clementson L. A. Boyd P. W. Trull T. W. (2020). Diel quenching of Southern Ocean phytoplankton fluorescence is related to iron limitation. Biogeosciences 17, 793–812.

[ref41] Schansker G. Tóth S. Z. Strasser R. J. (2006). Dark recovery of the Chl a fluorescence transient (OJIP) after light adaptation: the qT-component of non-photochemical quenching is related to an activated photosystem I acceptor side. Biochimica et Biophysica Acta (BBA)-Bioenergetics 1757, 787–797. doi: 10.1016/j.bbabio.2006.04.019, PMID: 16777056

[ref42] Schuback N. Hoppe C. J. M. Tremblay J. É. Maldonado M. T. Tortell P. D. (2017). Primary productivity and the coupling of photosynthetic electron transport and carbon fixation in the Arctic Ocean. Limnol. Oceanogr. 62, 898–921. doi: 10.1002/lno.10475

[ref43] Schuback N. Schallenberg C. Duckham C. Maldonado M. T. Tortell P. D. (2015). Interacting effects of Light and Iron availability on the coupling of photosynthetic Electron transport and CO 2 -assimilation in marine phytoplankton. PLoS One 10, 1–30. doi: 10.1371/journal.pone.0133235, PMID: 26171963 PMC4501554

[ref44] Schuback N. Tortell P. D. Berman-Frank I. Campbell D. A. Ciotti A. Courtecuisse E.. (2021). Single-turnover variable chlorophyll fluorescence as a tool for assessing phytoplankton photosynthesis and primary productivity: opportunities, Caveats and Recommendations’. Front. Mar. Sci. 8:895. doi: 10.3389/fmars.2021.690607

[ref45] Sezginer Y. Suggett D. J. Izett R. W. Tortell P. D. (2021). Irradiance and nutrient-dependent effects on photosynthetic electron transport in Arctic phytoplankton: A comparison of two chlorophyll fluorescence-based approaches to derive primary photochemistry. PLoS One 16, e0256410–e0256423. doi: 10.1371/journal.pone.0256410, PMID: 34882695 PMC8659313

[ref9006] Silsbe G. M. Oxborough K. Suggett D. J. Forster R. M. Ihnken S. Komárek O.. (2015). ‘Toward autonomous measurements of photosynthetic electron transport rates: An evaluation of active fluorescence-based measurements of photochemistry’, Limnol. Oceanogr, Methods 13, 138–155. doi: 10.1002/lom3.10014

[ref46] Suggett D. Moore M. Geider R. J. (2010). Estimating Aquatic Productivity from Active Fluorescence Measurement. Dordrecht: Springer Netherlands.

[ref47] Suggett D. J. Moore C. M. Hickman A. E. Geider R. J. (2009). Interpretation of fast repetition rate (FRR) fluorescence: sigatures of phytoplankton community structure versus physiological state. Mar. Ecol. Prog. Ser. 376, 1–19. doi: 10.3354/meps07830

[ref48] Triantaphylidès C. Havaux M. (2009). Singlet oxygen in plants: production, detoxification and signaling. Trends Plant Sci. 14, 219–228. doi: 10.1016/j.tplants.2009.01.008 19303348

[ref9007] Vihtakari M. Welcker J. Moe B. Chastel O. Tartu S. Hop H.. (2018). Black-legged kittiwakes as messengers of Atlantification in the Arctic. Scientific Reports 8:1178. doi: 10.1038/s41598-017-19118-8 29352216 PMC5775339

[ref49] Walczowski W. (2014). GeoPlanet: Earth and Planetary Sciences Atlantic Water in the Nordic Seas, Cham: Springer International Publishing.

[ref50] Walter B. Peters J. van Beusekom J. E. E. (2017). The effect of constant darkness and short light periods on the survival and physiological fitness of two phytoplankton species and their growth potential after re-illumination. Aquat. Ecol. 51, 591–603. doi: 10.1007/s10452-017-9638-z

[ref51] Xu K. Lavaud J. Perkins R. Austen E. Bonnanfant M. Campbell D. A. (2018). Phytoplankton σPSII and excitation dissipation; implications for estimates of primary productivity. Front. Mar. Sci. 5. doi: 10.3389/fmars.2018.00281

