# Supplementary material for: Fluorescence-based primary productivity estimates are influenced by non-photochemical quenching dynamics in Arctic phytoplankton
Source: Front Microbiol. 2023 Dec 7;14:1294521. doi: 10.3389/fmicb.2023.1294521 (PMC10741645; doi:10.3389/fmicb.2023.1294521)
Supplement: Supplementary file 1 [file Table_1.DOCX]

Supplementary Material

Fluorescence-based primary productivity estimates are influenced by non-photochemical quenching dynamics in Arctic phytoplankton

Yayla Sezginer^*^, Douglas Campbell, Sacchinandan Pillai, Philippe Tortell

*** Correspondence:** Yayla Sezginer: [ysezginer@eoas.ubc.ca](mailto:ysezginer@eoas.ubc.ca)

# Primary and secondary ChlF parameters used in this study and their definitions.

# Table S1. Primary parameters were derived by fitting the biophysical model (Kolber et al., 1998) to each ChlF transient. Secondary parameters were derived from primary parameters as described by Tortell et al. (2023). The prime notation indicates parameters measured in the presence of actinic light.

| Term | Definition | Units |
| --- | --- | --- |
| **Primary parameters** |  |  |
| $F_{o}$, $F_{o}^{''}$_,_ Fʹ | Minimum ChlF measured in NPQ- relaxed, semi-relaxed, and light-regulated states, respectively | Relative Units |
| $F_{m}$, $F_{m}^{''}$_,_ Fʹ, | Maximum ChlF representative of a closed RCII pool in NPQ- relaxed, semi-relaxed, and light-regulated states, respectively | Relative Units |
| $\sigma_{PSII}, \sigma_{PSII}^{''}, \sigma_{PSII}^{'}$ | PSII functional absorption cross-section in NPQ- relaxed, semi-relaxed, and light-regulated states, respectively | Å^2^ PSII^-1^ |
| *F*_v_/*F*_m_, *F*ʹʹ_v_/*F*ʹʹ_m_ | Maximum photochemical yield in the NPQ relaxed and semi-NPQ relaxed state; (F_m_-F_o_)/F_m_ | Dimensionless |
| *F*_q_ʹ/*F*_m_ʹ | Light regulated photochemical yield (F_m_ʹ - Fʹ)/ F_m_ʹ | Dimensionless |
| *F*_q_ʹ/*F*_v_ʹ | Fraction of functional RCIIs in the open state (F_m_ʹ - Fʹ)/ (F_m_ʹ - F’_o_) | Dimensionless |
| **Secondary parameters** |  |  |
| qN | Coefficient of NPQ; $1-F_{v}^{'}/F_{v}$ = qE + qT + qI | Dimensionless |
| qE | ‘Fast’ relaxing NPQ regulated by lumen pH | Dimensionless |
| qZ | Quenching by photoprotective xanthophyll forms | Dimensionless |
| qT | State transitions regulated by protein phosphorylation at light harvesting complexes | Dimensionless |
| qI | ‘Slow’ NPQ due to photoinhibition, regulated by protein repair | Dimensionless |
| $\tau_{qE},\tau_{qT},\tau_{qZ}, \tau_{qI}$ | Half-life time constants of qE, qT, qZ, and qI relaxation, respectively | s, min, or hrs |
| ETR | PSII electron transport rate | e^-^ s^-1^ RCII^-1^ |

# Taxonomic compositional data collected on-station in the Canadian Arctic Archipelago

**Table S2.** Chemtax results derived from pigment samples is expressed as fractional abundance. < dl symbolizes below detection limit.

| Station | Diat. | Dino. | c3-flag | Crypt | Chryso | Hapto | Pras-2 | Pras-3 | Chloro |
| --- | --- | --- | --- | --- | --- | --- | --- | --- | --- |
| **Surface** |  |  |  |  |  |  |  |  |  |
| KA1 | < dl | 0.02 | < dl | 0.07 | 0.01 | 0.80 | < dl | < dl | 0.10 |
| KA3 | 0.19 | 0.10 | 0.04 | 0.09 | 0.07 | 0.08 | < dl | 0.37 | 0.06 |
| KA5 | 0.18 | 0.13 | 0.04 | 0.06 | 0.08 | 0.40 | 0.06 | 0.04 | < dl |
| KB5 | < dl | 0.11 | 0.19 | 0.05 | 0.07 | 0.01 | < dl | 0.56 | < 0.01 |
| KB3 | 0.12 | 0.08 | 0.21 | 0.08 | 0.04 | 0.04 | < dl | 0.42 | < dl |
| KB1 | < dl | 0.05 | 0.03 | 0.10 | 0.06 | 0.63 | < dl | 0.03 | 0.10 |
| KC5 | 0.15 | 0.12 | 0.06 | 0.08 | 0.04 | 0.09 | < dl | 0.42 | 0.03 |
| KC3 | 0.30 | 0.06 | 0.05 | 0.07 | 0.05 | 0.09 | 0.06 | 0.32 | < dl |
| KC1 | < dl | 0.04 | 0.24 | 0.02 | 0.02 | 0.67 | < dl | < dl | 0.01 |
| KD1 | 0.03 | 0.06 | < dl | 0.09 | 0.04 | 0.69 | < dl | 0.05 | 0.04 |
| KD3 | 0.13 | 0.10 | < 0.01 | 0.08 | 0.07 | 0.22 | < dl | 0.36 | 0.03 |
| KD5 | 0.04 | 0.10 | 0.02 | 0.09 | 0.08 | 0.14 | 0.02 | 0.46 | 0.05 |
| CLYDE | 0.03 | 0.06 | 0.04 | 0.08 | 0.07 | 0.14 | 0.04 | 0.54 | 0.05 |
| N324 | 0.18 | 0.13 | 0.20 | 0.05 | 0.06 | 0.04 | < dl | 0.33 | < dl |
| N323 | 0.26 | 0.15 | 0.24 | 0.05 | 0.04 | 0.05 | < dl | 0.22 | < dl |
| N103 | 0.04 | 0.08 | 0.16 | 0.16 | 0.07 | 0.14 | 0.01 | 0.32 | 0.03 |
| N105 | 0.34 | 0.09 | 0.09 | 0.09 | 0.05 | 0.09 | 0.04 | 0.21 | < 0.01 |
| N110 | 0.07 | 0.10 | 0.28 | 0.12 | 0.03 | 0.07 | < dl | 0.32 | 0.01 |
| N116 | < dl | 0.09 | 0.08 | 0.10 | 0.09 | 0.12 | 0.01 | 0.51 | < dl |
| **Subsurface** |  |  |  |  |  |  |  |  |  |
| KA1 | < dl | < dl | < dl | 0.04 | < dl | 0.66 | < dl | 0.25 | 0.06 |
| KA3 | 0.34 | 0.13 | 0.32 | 0.07 | 0.03 | 0.03 | 0.06 | < dl | 0.02 |
| KA5 | 0.01 | 0.12 | 0.37 | 0.14 | 0.07 | 0.16 | 0.06 | < dl | 0.06 |
| KB5 | < dl | 0.11 | 0.05 | 0.09 | 0.08 | 0.21 | 0.05 | 0.41 | < 0.01 |
| KB3 | 0.19 | 0.16 | 0.37 | 0.07 | 0.04 | 0.05 | < 0.01 | 0.11 | 0.01 |
| KB1 | < dl | 0.09 | 0.42 | 0.08 | 0.02 | 0.30 | 0.02 | < dl | 0.07 |
| KC5 | < dl | 0.14 | 0.14 | 0.05 | 0.11 | 0.43 | 0.02 | 0.04 | 0.06 |
| KC3 | 0.54 | 0.07 | 0.34 | 0.03 | < dl | < 0.01 | 0.01 | < dl | 0.01 |
| KC1 | < dl | < dl | < dl | 0.02 | < dl | 0.84 | 0.01 | 0.11 | 0.03 |
| KD1 | < dl | 0.05 | < dl | 0.03 | 0.06 | 0.66 | < dl | 0.18 | 0.01 |
| KD3 | 0.50 | 0.08 | 0.30 | 0.04 | 0.01 | < dl | 0.01 | 0.07 | < dl |
| KD5 | 0.62 | 0.04 | 0.27 | 0.02 | < dl | < dl | 0.02 | 0.03 | < 0.01 |
| CLYDE | < dl | 0.10 | 0.14 | 0.10 | 0.06 | 0.12 | 0.08 | 0.37 | 0.04 |
| N324 | 0.53 | 0.08 | 0.31 | 0.03 | < dl | < dl | 0.05 | < 0.01 | < dl |
| N323 | 0.12 | 0.12 | 0.50 | 0.03 | 0.01 | < dl | 0.02 | 0.20 | < dl |
| N103 | 0.43 | 0.08 | 0.12 | 0.12 | 0.02 | 0.07 | 0.08 | 0.08 | < dl |
| N105 | 0.15 | 0.12 | 0.02 | 0.18 | 0.07 | 0.21 | 0.23 | 0.02 | < dl |
| N110 | < dl | 0.11 | 0.09 | 0.18 | 0.06 | 0.16 | < 0.01 | 0.35 | 0.05 |
| N116 | < dl | 0.09 | 0.03 | 0.10 | 0.08 | 0.16 | 0.11 | 0.44 | < dl |

# Ancillary oceanographic data for Canadian Arctic station surface samples

| Station | Sample Depth (m) | Lat (^o^N) | Lon (^o^E) | MLD (m) | Chl (mg m^-3^) | NO3 ($\mu$M) | PP:PS |
| --- | --- | --- | --- | --- | --- | --- | --- |
| KA1 | 1 | 66.61 | -61.19 | 19 | 0.34 | 4.1 | 0.21 |
| KA3 | 1 | 66.73 | -59.61 | 15 | 0.58 | < dl | 0.43 |
| KA5 | 1 | 66.88 | -57.96 | 28 | 1.46 | < dl | 0.34 |
| KB5 | 1 | 67.59 | -59.02 | 21 | 0.70 | 0.01 | 0.36 |
| KB3 | 1 | 67.33 | -60.28 | 19 | 0.39 | < dl | 0.44 |
| KB1 | 1 | 67.06 | -61.51 | 38 | 0.18 | 0.07 | 0.24 |
| KC5 | 1 | 68.15 | -59.97 | 21 | 0.65 | < 0.01 | 0.47 |
| KC3 | 1 | 67.75 | -61.27 | 13 | 0.25 | < dl | 0.29 |
| KC1 | 1 | 67.35 | -62.53 | 27 | 0.22 | < dl | 0.15 |
| KD1 | 1 | 67.47 | -63.69 | 10 | 0.21 | < dl | 0.18 |
| KD3 | 1 | 68.24 | -62.60 | 20 | 0.62 | < dl | 0.36 |
| KD5 | 1 | 69.00 | -61.41 | 14 | 0.35 | 0.29 | 0.32 |
| CLYDE | 1 | 70.35 | -68.45 | 6 | 0.42 | < dl | 0.34 |
| N324 | 1 | 73.83 | -79.60 | 26 | 0.82 | 0.03 | 0.22 |
| N323 | 1 | 74.15 | -79.30 | 25 | 0.90 | < dl | 0.23 |
| N103 | 1 | 76.18 | -76.98 | 81 | 0.31 | 5.39 | 0.26 |
| N105 | 1 | 76.12 | -75.76 | 18 | 0.21 | 3.96 | 0.23 |
| N110 | 1 | 76.30 | -73.62 | 58 | 0.46 | 4.23 | 0.27 |
| N116 | 1 | 76.38 | -70.52 | 9 | 0.70 | 0.25 | 0.29 |

# Ancillary oceanographic data for Canadian Arctic station subsurface samples

| Station | Sample Depth (m) | Lat (^o^N) | Lon (^o^E) | MLD (m) | Chl (mg m^-3^) | NO3 ($\mu$M) | PP:PS |
| --- | --- | --- | --- | --- | --- | --- | --- |
| KA1 | 19 | 66.61 | -61.19 | 19 | 0.55 | 4.1 | 0.21 |
| KA3 | 24 | 66.73 | -59.61 | 15 | 8.1 | < dl | 0.15 |
| KA5 | 27 | 66.88 | -57.96 | 28 | 3.4 | < dl | 0.20 |
| KB5 | 22 | 67.59 | -59.02 | 21 | 1.07 | 0.31 | 0.27 |
| KB3 | 27 | 67.33 | -60.28 | 19 | 6.8 | 0.39 | 0.17 |
| KB1 | 40 | 67.06 | -61.51 | 38 | 0.53 | 0.03 | 0.14 |
| KC5 | 25 | 68.15 | -59.97 | 21 | 4.4 | 0.14 | 0.16 |
| KC3 | 23 | 67.75 | -61.27 | 13 | 3.7 | < dl | 0.12 |
| KC1 | 25 | 67.35 | -62.53 | 27 | 0.54 | < dl | 0.16 |
| KD1 | 40 | 67.47 | -63.69 | 10 | 0.35 | < dl | 0.14 |
| KD3 | 24 | 68.24 | -62.60 | 20 | 4.8 | 0.24 | 0.13 |
| KD5 | 22 | 69.00 | -61.41 | 14 | 6.1 | 0.83 | 0.11 |
| CLYDE | 10 | 70.35 | -68.45 | 6 | 0.45 | < dl | 0.24 |
| N324 | 35 | 73.83 | -79.60 | 26 | 2.4 | 0.04 | 0.17 |
| N323 | 27 | 74.15 | -79.30 | 25 | 1.6 | < dl | 0.16 |
| N103 | 35 | 76.18 | -76.98 | 81 | 0.24 | 4.3 | 0.22 |
| N105 | 45 | 76.12 | -75.76 | 18 | 0.36 | 6.1 | 0.24 |
| N110 | 17 | 76.30 | -73.62 | 58 | 0.48 | 5.7 | 0.30 |
| N116 | 5 | 76.38 | -70.52 | 9 | 0.72 | < dl | 0.25 |
